# Universal Exposition

**Published:** 1904-04

**Authors:** 


### UNIVERSAL EXPOSITION.

### Fourth International Dental Congress, August 29 to September 3, 1904, Saint Louis.

We, the Committee on Local Information, submit the following facts, important for the success of the Fourth International Dental Congress:

Every member of the profession who has at heart the welfare of dentistry should give it support, financially and by action.

We, as American members of the dental profession, would well keep in mind that the Congress will be held in our great country, and pride should prompt us to action to make the meeting one of great magnitude and far excelling its predecessors.

Let every dentist take hold with a will and push it to a successful issue.

This Congress should be of great interest to all of the profession. There is planned a monster clinic from the best clinicians of the world, scientific papers from the pens of the most noted and scholarly men of the profession, and an exhibit to excel all others.

The meetings, the section meetings, the clinics and the exhibits will be held in the great Coliseum, where ample accommodations are secured.

Hotel Jefferson, the general headquarters of the Dental Congress, is one of the most fashionable and complete hotels in the West, if not in America, and is located on Twelfth street, one block from the Coliseum. Hotels of Saint Louis will be sufficient to meet all requirements.

The Information Bureau of the Exposition has a list of ninety-seven well established hotels now doing business in Saint Louis, with a capacity of 41,000 guests, at prices ranging from fifty cents a day up on the European plan and from one dollar a day up on the American plan. These established hotels have been supplemented during the year 1903 by thirty-five new permanent hotels now opening, or about to open, increasing the permanent hotel capacity to 67,000 guests, at prices ranging from one dollar a day up. The Exposition management holds the signed agreement of the leading hotels that "Rates shall not be increased during the World's Fair period." Prices are now lower in Saint Louis than in any other city for similar hotel accommodations and service.

The Exposition Information Bureau's list of 132 permanent hotels includes only the better class. There are now 173 hotels, large and small, in operation in the city, and the new hotel enterprises being inaugurated justify the belief that the number will reach 250 before the opening day of the World's Fair.

Besides hotels with accommodations for more than 200,000 guests, the Exposition Information Bureau has a list of boarding-houses and rooming-houses of respectable character, on the street car lines, with lodgings for 65,000 guests, and a list of private houses that will let rooms for 20,000 persons.

All over the city, apartment houses and rooming-houses are available for those who prefer rooms away from crowds, with meals at the restaurants.

There are 485 restaurants in Saint Louis that have a national reputation for good fare, good service, cleanliness and moderate prices. Twenty of these 485 restaurants can take care of 40,000 patrons.

The climate of Saint Louis is temperate in summer and most delightful in the spring and fall. It is the most central and accessible of the four large cities of the United States, twenty-seven railways entering it, and passenger steamers on the Mississippi reaching it from North and South.

World's Fair cheap rates on railways and steamboats will be offered during the whole Exposition season, as follows:

RATES ADOPTED BY THE FOLLOWING ASSOCIATIONS:

New England Passenger Association, Trunk Line Association, Central Passenger Association, and the Southeastern Passenger Association.

Tickets will be on sale from April 15th.

Season tickets for eighty per cent. of double one fare, good to return until December 15th.

For sixty days, one and one-third fares, not good to return after December 15th.

For ten days, one fare, plus $2.00, from points within two hundred and fifty (250) miles of Saint Louis.

For fifteen days, one fare, plus $2.00, from points over two hundred and fifty miles from Saint Louis.

Saint Louis is the fourth city of the United States in point of population, having 750,000 people. Certainly no city is more attractive with interest for the student of nature, science, history, etc. There are twenty-four public parks, containing over 2,100 acres, all well improved.

The World's Fair grounds lie five miles from the river, on the western edge of the city, and are reached quickly and comfortably by steam railway and fast trolley lines.

The visitors reach the city through the largest and most meautiful railway station in the world. Thirty-two tracks run into the station side by side, and the midway, or glass-roofed hall in front of the gates to the trains, will hold 30,000 people. Most of the hotels, except those temporary ones near the World's Fair grounds, are within ten minutes' ride of the station, and the heart of the business district. Street cars, reaching all of the hotels for one fare, pass the station, and the cab, carriage and baggage system is excellent.

The great Music Hall and Coliseum building that is secured for the meetings of the International Dental Congress is downtown, on Olive street, between Thirteenth and Fourteenth streets, within easy walking distance of Union Station, the hotels and the business district. It has a seating capacity for 8,000 people, with section rooms that will be arranged for the various committees and exhibitors.

Naturally, every member attending the Fourth International Dental Congress will wish to see the World's Fair. This will be the most magnificent the world has ever seen, and it will probably be the last great exposition to be held in this country for many years, because of the enormous expenditure of labor and money attending it.

Congressman Bartholdt, in a recent speech made before Congress, said; "The Universal Exposition at Saint Louis is the apotheosis of centuries of civilization. It is the culminating perfection of those wonderful international spectacles which have served to impress on our minds that it is good to be a living participant in the glories of this world.

"A decade of human achievement has elapsed since the Columbian pageantry of progress at Chicago. Every American who saw the 'White City' thrilled with the thought that the nations of the earth had assembled in the greatest Republic to do homage to the genius of enlightenment.

"Triumphs of the Emperors of Imperial Rome were but the mock pomp of childish fancies compared to the triumphs of peace as celebrated by such a labor of love at Saint Louis. On the May Day of this year the gates of welcome will be flung wide open and the vision of the century will then unfold its prophetic beauty for the uplifting of humanity.

"All in all, the Universal Exposition of 1904 will be the sensational climax of the twentieth century, the grandest victory of peace and civilization, the greatest triumph human genius has yet achieved. To millions of its visitors it will be an academy of learning, an inspiration and an inexhaustible source of genuine delight, and the memories of the 'Ivory City' will live and bear fruit in ages yet to come."

We appeal to all reputable, legally qualified practitioners of dentistry to interest themselves in the success of this great international meeting.

*Let every dentist in America put forth his greatest effort to advance the cause. Make application at once through your state chairman for a membership certificate. See that your fellow practitioner is enrolled, and then, and not till then, will you have done your full duty.*

The membership fee to the Congress will be $10.00. It will entitle the holder to the official badge and all the rights and privileges of the Congress. He will also receive one copy of the transactions. Without a membership certificate it will be impossible to get into the general meetings, sections or clinics, or attend any of the various entertainments given during the Congress.

This great Congress presents to the American dentist an opportunity to see and hear the brightest and most learned men of the profession from all parts of the world, men of international reputation that shine as clinicians, and men of great renown of the inventive turn of mind. This International meeting will be the Mecca of the profession of the world. We will all receive new light and be stimulated to a higher appreciation of our noble profession.

The local Committee of Arrangements and Reception, with its various minor committees, is working with increasing energy and will be ready to meet you and extend you a most hearty welcome.

Any further information can be obtained from the Committee.

D. O. M. LeCron,
Chairman, Missouri Trust Bldg.
Max Fendler,
Secretary, Missouri Trust Bldg.
George H. Gibson,
H. F. D'Oench,
G. L. Kitchen,
S. H. Voyles,
Orem H. Manhard,
Joseph G. Pfaff,
Permanent Local Committee and
Bureau of Information.

